# Mitochondrial transplantation for therapeutic use

**DOI:** 10.1186/s40169-016-0095-4

**Published:** 2016-04-29

**Authors:** James D. McCully, Sidney Levitsky, Pedro J. del Nido, Douglas B. Cowan

**Affiliations:** Division of Cardiac Surgery, Boston Children’s Hospital, 300 Longwood Ave., Enders Building, EN 407, Boston, MA 02115 USA; Division of Cardiac Surgery, Beth Israel Deaconess Medical Center, 110 Francis Street, Suite 2A, Boston, MA 02115 USA; Department of Anesthesiology, Perioperative and Pain Medicine, Boston Children’s Hospital, 300 Longwood Ave., Endres Building, EN 312, Boston, MA 02115 USA; Harvard Medical School, Boston, MA USA

**Keywords:** Ischemia/reperfusion injury, Mitochondria, Myocardium, Surgery

## Abstract

Mitochondria play a key role in the homeostasis of the vast majority of the body’s cells. In the myocardium where mitochondria constitute 30 % of the total myocardial cell volume, temporary attenuation or obstruction of blood flow and as a result oxygen delivery to myocardial cells (ischemia) severely alters mitochondrial structure and function. These alterations in mitochondrial structure and function occur during ischemia and continue after blood flow and oxygen delivery to the myocardium is restored, and significantly decrease myocardial contractile function and myocardial cell survival. We hypothesized that the augmentation or replacement of mitochondria damaged by ischemia would provide a mechanism to enhance cellular function and cellular rescue following the restoration of blood flow. To test this hypothesis we have used a model of myocardial ischemia and reperfusion. Our studies demonstrate that the transplantation of autologous mitochondria, isolated from the patient’s own body, and then directly injected into the myocardial during early reperfusion augment the function of native mitochondria damaged during ischemia and enhances myocardial post-ischemic functional recovery and cellular viability. The transplanted mitochondria act both extracellularly and intracellularly. Extracellularly, the transplanted mitochondria enhance high energy synthesis and cellular adenosine triphosphate stores and alter the myocardial proteome. Once internalized the transplanted mitochondria rescue cellular function and replace damaged mitochondrial DNA. There is no immune or auto-immune reaction and there is no pro-arrhythmia as a result of the transplanted mitochondria. Our studies and those of others demonstrate that mitochondrial transplantation can be effective in a number of cell types and diseases. These include cardiac and skeletal muscle, pulmonary and hepatic tissue and cells and in neuronal tissue. In this review we discuss the mechanisms leading to mitochondrial dysfunction and the effects on cellular function. We provide a methodology for the isolation of mitochondria to allow for clinical relevance and we discuss the methods we and others have used for the uptake and internalization of mitochondria. We foresee that mitochondrial transplantation will be a valued treatment in the armamentarium of all clinicians and surgeons for the treatment of varied ischemic disorders, mitochondrial diseases and related disorders.

## Introduction

Mitochondria are unique organelles containing their own DNA that encodes the mitochondrial subunits of the oxidative phosphorylation system. The role of the mitochondria has primarily been considered to be that of energy production and synthesis through fermentation and glycolysis with most of the energy requirements of the cell being derived through the electron transport chain and oxidative phosphorylation [1].

In the myocardium mitochondria constitute 30 % of the total myocardial cell volume and play an important role in the maintenance and modulation of organ function. The mitochondria provide for the majority of cellular energy to the heart and are dependent upon a continuous supply of oxygen delivered by the coronary arteries to maintain the energy requirements of the myocardial cells. The attenuation or cessation of coronary blood flow decreases oxygen delivery to the myocardium (myocardial ischemia) and induces a cascade of cellular events which rapidly alter mitochondrial function leading to myocardial cellular dysfunction and/or cell death and myocardial contractile functional impairment that extends after the restoration of coronary blood flow to the heart [2–9].

Herein we will review the role of the mitochondria in the myocardium following myocardial ischemia and provide evidence to show that the augmentation or replacement of mitochondria is efficacious. To this end, we have proposed a robust therapeutic intervention to ameliorate myocardial ischemia and significantly decrease morbidity and mortality in the myocardium following ischemia, by direct injection of autologous mitochondria, isolated from non-ischemic tissue obtained from the patient’s own body and then directly injected into the ischemic myocardium. In this review we discuss the rationale and efficacy of mitochondrial transplantation and provide evidence for alternative usage.

## Review

A significant body of evidence now exists to show that in addition to high energy synthesis, the mitochondrion plays an important role in regulating a variety of diseases and genetic disorders [2]. In a series of studies using animal models we and others have demonstrated that ischemia occurring through decreased blood flow to the myocardium significantly alters myocardial mitochondrial structure and function [3–9]. These alterations while occurring during ischemia persist after the restoration of blood flow and oxygen delivery to the heart and are associated with significantly decreased myocardial function and myocardial cell survival [3–6].

The mechanisms for these alterations in function are varied. In studies extending over 25 years, we have shown that ischemia significantly increases myocardial cytoplasmic calcium accumulation and that this increase results in significantly increased mitochondrial calcium accumulation and significantly increases mitochondrial volume [3–7]. The mechanism modulating increased mitochondrial volume is directly related to mitochondrial calcium handling. Under homeostatic conditions, the mitochondrial inner membrane containing the electron transport chain expels protons to the cytosol, creating a charge gradient that provides the passive energy for calcium influx to the mitochondrion by the calcium uniporter. Following ischemia, the increased cytosolic calcium accumulation is taken up in the mitochondrion. The increase in mitochondrial calcium destabilizes the inner mitochondrial membrane, and causes the inner membrane pore to open and permit further cation movement. This process is referred to as “futile calcium cycling”, an energy dependent process requiring high energy phosphates (ATP) to transport calcium against the electrochemical gradient out of the mitochondrion [3–6]. As calcium enters the mitochondria, water follows leading to mitochondrial matrix swelling and eventually mitochondrial rupture and myocardial cell dysfunction [7].

**Fig. 1:**
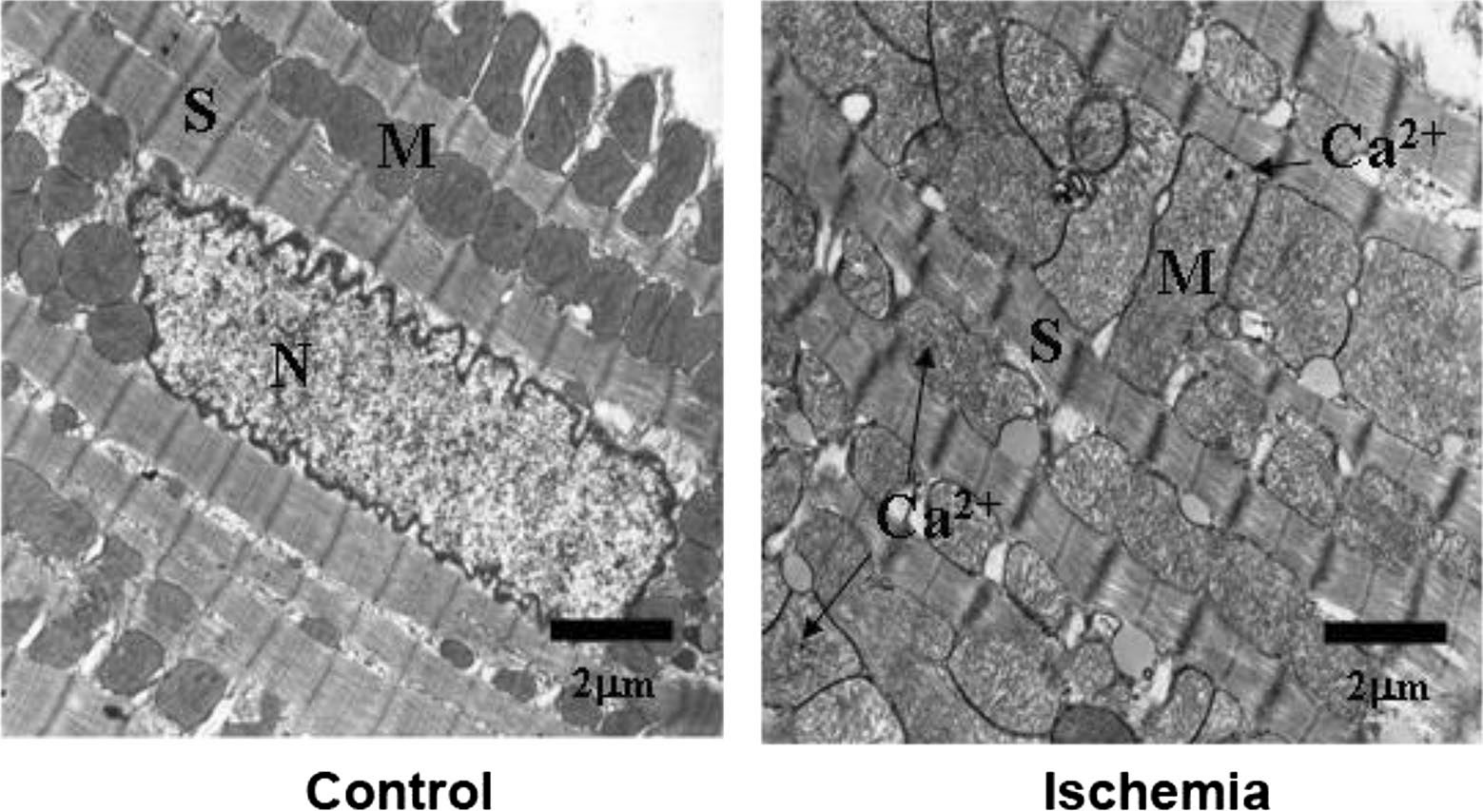
Mitochondrial damage following ischemia. Representative transmission electron photomicrographs from left ventricular tissue from *Control* (non-ischemic) and *Ischemia* (30 min ischemia) hearts. In *Control* hearts sarcomere structure is preserved and mitochondria have electron dense intracristae matrix and normal size. In *Ischemia* hearts there is separation myofilaments and severely swollen and electron transparent mitochondria containing numerous mitochondrial calcium granules. Calcium granules (*Ca* ^*2+*^), mitochondrion (*M*), sarcomere (*S*) and nucleus (*N*) are indicated. *Scale bars* (2 um are shown)

In the isolated perfused heart and in the in vivo pig and sheep model of regional and global ischemia and reperfusion, we and others have shown that following 20–30 min of myocardial ischemia the matrix and cristae of mitochondria in the heart are severely swollen (Fig. 1) [6–8].

It has been estimated that greater than 88 % of myocardial mitochondria are swollen following 20–30 min of ischemia and exhibit increased intermembrane space and swollen cristae and disrupted matrix [6, 9]. We have confirmed these changes in mitochondrial structure using both transmission electron microscopy and the light scattering technique [6, 9]. Transmission electron microscopy of greater than 500 mitochondria from each tissue source revealed that the ischemic myocardial mitochondria are electron translucent and swollen as compared to mitochondria from non-ischemic areas [6].

Light scattering was also used to determine changes in mitochondrial volume. The light scattering technique is sensitive to changes in the intermembrane space and the shape of mitochondria, premised upon the observation that swollen mitochondria are associated with a decrease in light absorbance. Mitochondrial matrix volume was measured as the decrease in absorbance at 520 nm, the isosbestic point for the mitochondrial cytochromes, allowing for the estimation of volume insensitive to changes in redox state [6]. Our studies using light scattering demonstrated that ischemic myocardial mitochondria are swollen as compared to non-ischemic mitochondria [6].

These alterations in mitochondrial structure have been proposed to play a leading role in diminished mitochondrial oxygen consumption and high energy synthesis. Under normoxic conditions the electron transport chain allows for the active transport of hydrogen ions across the inner mitochondrial membrane, out of the matrix to the intermembrane space, creating an electrochemical gradient [10]. This gradient provides the energy that allows the release of ATP from ATP synthase and export to the cytosol. This export is regulated by the ATP/ADP translocator (ANT), mitochondrial creatine kinase (Mi-CK) and the voltage dependent anion channel (VDAC) [10]. The space between the outer and the inner mitochondrial membrane is bridged by Mi-CK in close contact with VDAC and ANT. The close association between VDAC, ANT and MI-CK confers a low permeability to nucleotides and requires a narrow intermembrane distance [11]. It has been proposed that mitochondrial swelling would increase intermembrane distance and dissociate the functional interaction between VDAC, ANT and MI-CK thus compromising efficient energy transfer during reperfusion and decrease high energy synthesis in the myocardium [11].

Our studies in large and small animal have also shown that following ischemia, myocardial mitochondrial oxygen consumption is significantly decreased [6.8]. Mitochondrial function assessed by oximetry demonstrated that state three oxygen consumption and respiratory control index in malate (complex I substrate) and succinate (complex II substrate) energized myocardial mitochondria were significantly decreased (p < 0.05) as compared to control myocardial mitochondria which did not undergo ischemia [6, 8].

One enzyme which may be an indicator of impaired mitochondrial function is cytochrome oxidase. Cytochrome oxidase is the terminal enzyme complex of the inner mitochondrial electron transport chain and has been shown to be vital in the production of high energy phosphate. Our studies have shown that following ischemia cytochrome oxidase mRNA levels and enzyme activity (Vmax) is significantly decreased [4, 5]. This decrease in cytochrome oxidase would contribute to the diminishment of high energy phosphates.

Support for this mechanism comes from ^31^P nuclear magnetic resonance (NMR) studies in the isolated perfused rabbit heart where we have shown that ischemia induces a significant decrease in phosphocreatine, inorganic phosphate, high energy stores, and high energy synthesis [12]. The decrease in high energy stores needed for myocardial hemodynamic function occurs during ischemia and persists after blood flow and oxygenation of the myocardial tissue is re-established [12]. Our studies demonstrate that high energy levels are significantly decreased to 36 ± 3 % of control following 30 min ischemia and remain significantly decreased at 15 min after the restoration of blood flow and oxygenation of myocardial tissue is re-established [12]. All these events are associated with significantly decreased myocardial function [12].

Ischemia also affects the mitochondrial genome and mitochondrial transcript and protein synthesis. In human studies we have shown that myocardial ischemia results in significant damage to the mitochondrial genome [13]. Using primer-shift PCR analysis of human tissue samples we demonstrated that following ischemia there is a large mitochondrial DNA (mtDNA) deletion (approximately 7.3 kb) that encompasses the region encoding 11 electron transport protein subunits [13]. These include 5 of the 7 protein subunits of complex I (ND3, ND4, ND4L, ND5, ND6), the one mitochondrial encoded protein subunit in complex III (cyt b), 1 of the 3 protein subunits in complex IV (cytochrome oxidase III) and 1 of the 3 subunits in complex V (ATPase 6) [12]. The mtDNA breakpoints associated with this deletion are located in the ATP synthase eight gene and in the cytochrome b gene and are flanked by four direct repeat sequences. This mtDNA region has been previously reported to be a major hotspot for deletion in the rat [14]. Proximal to the 5′ deletion breakpoint is a 13 bp sequence (located between nucleotides 8470–8482) that has been previously identified to be associated with the mitochondrial disorders of Kearns-Sayre syndrome, chronic progressive external ophthalmoplegia and Leber’s hereditary optic neuropathy, suggesting that this is hot-spot may be of importance in the onset and disability associated with mitochondrial associated genetic diseases and syndromes [14]. Interestingly, the 7.3 kb mtDNA deletion is apparent in patients with clinical indications of poor recovery following cardiac surgery suggesting that mtDNA deletions may provide an important indicator to surgical outcome in the cardiac surgical patient population [14].

These changes also affect the nuclear and mitochondrial transcript and proteome. Previous studies have shown that there is a close association between nuclear and mitochondrial transcription and the nuclear and mitochondrial proteome [15]. This association is apparent with the interrelated actions of nuclear and mitochondrial transcription and translation of the mitochondrial electron transport chain.

The human mitochondrial genome is a 16,569 base pair circular molecule, with approximately 4.6 copies per mitochondrion, consisting of two strands, a guanine-rich strand, the heavy strand (H-strand) and a cytosine-rich strand, the light strand (L-strand). The heavy strand encodes 28 genes, and the light strand encodes night genes. These genes include the coding regions for 12S and 16S rRNA, 22 tRNAs, and the 13 hydrophobic proteins of the electron transport chain [1].

The mitochondrial electron transport chain consists of five tightly linked tandem associated complexes (complexes I–V) residing within the mitochondrial inner membrane. The complexes of the electron transport chain consist of 76 protein subunits. Thirteen of these 76 protein subunits are encoded by mtDNA and include seven protein subunits in complex I (ND1, ND2, ND3, ND4, ND4L, ND5, ND6); 1 subunit in complex III (cytochrome *b*); three subunits in complex IV (cytochrome oxidases I, II, III); and three subunits in complex V (ATPase 6, 8).

Complex II, coenzyme Q_10_, cytochrome c and the vast majority of the remaining protein subunits of the electron transport chain are encoded by nuclear DNA [1].

In animal studies, we have found that myocardial ischemia induces significant alterations in nuclear and mitochondrial transcriptomic and proteomic expression levels. In the ischemia/reperfusion rabbit heart model, functional annotation clustering analysis using an enrichment score <2.0 and P > 0.05, revealed down-regulation of mitochondrion function and energy production, cofactor catabolism, generation of precursor metabolites of energy, cellular carbohydrate metabolism, regulation of biosynthesis, as well as the regulation of transcription and mitochondrial structure and function [16]. All these changes are associated with significantly decreased myocardial function and decreased myocardial cell viability.

## A role for mitochondrial transplantation

In summary, our studies demonstrate that following the onset of myocardial ischemia there are alterations in mitochondrial volume [6], function [6, 8], mitochondrial calcium accumulation [4, 5, 12], mitochondrial enzyme activity (cytochrome oxidase) [5], mitochondrial complex activity [16], high energy synthesis [6, 8, 12], mtDNA [13], the mitochondrial modulated intrinsic cell death pathway [17, 18] and mitochondrial transcriptomics and proteomics [16, 18, 19]. All these deleterious events occur during ischemia and persist subsequent to the restoration of tissue reperfusion, resulting in severely compromised post-ischemic functional recovery and diminished cellular viability [4–8, 12, 13, 16–19].

**Fig. 2:**
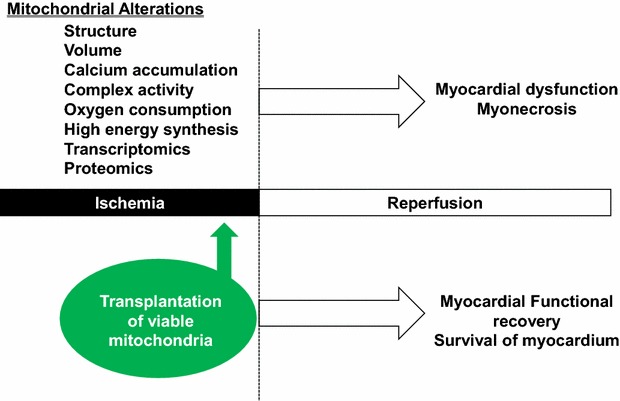
Myocardial mitochondrial changes occurring during ischemia. The changes to mitochondria occurring during ischemia are noted. These changes extend following the restoration of blood flow and oxygen delivery to the myocardium (reperfusion) and significantly decrease myocardial function and cell viability. Mitochondrial transplantation (the direct injection of viable exogenous mitochondria) into the ischemic myocardium, just prior to reperfusion significantly enhances myocardial function and cell viability

Based on these events occurring during myocardial ischemia and reperfusion (Fig. 2) we reasoned that the augmentation or replacement of mitochondria altered or damaged during ischemia with intact viable mitochondria may allow for myocardial cell rescue. We speculated that mitochondria isolated from a non-ischemic area in the patient’s own body and then transplanted into the ischemic region would provide a means to supplement the mitochondrial functions injured during ischemia and allow for myocardial recovery [19, 20].

### Mitochondria isolation and purification

To allow for clinical use of mitochondria for transplantation, the isolation and purification of mitochondria must be timely. Most cardiac surgical procedures last 40 min to several hours in duration. In the longer procedures, established methods for isolating mitochondria, which generally take >90 min, may be adequate [21–26]. However, for practical usage in procedures taking less than an hour, these long duration isolation procedures are impractical.

**Fig. 3:**
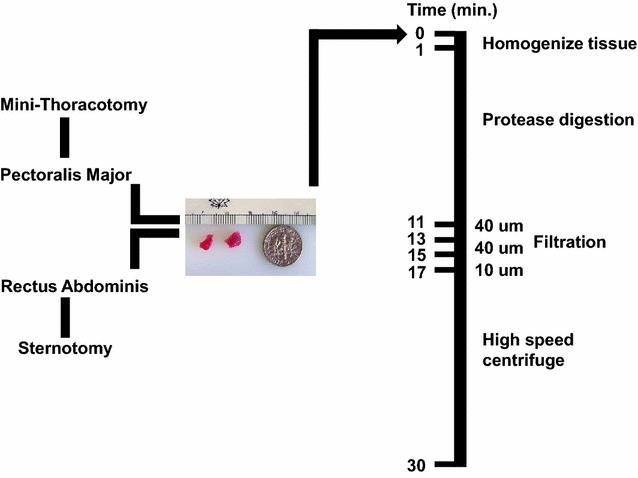
Mitochondrial isolation. Our protocol for mitochondrial isolation is illustrated. In brief a mini-thoracotomy or a sternotomy is performed and tissue is obtained from either pectoralis major or from rectus abdominous muscle. The amount of tissue is small and is shown in comparison to an American 10 cent piece. Mitochondria are isolated. Our methodology for mitochondrial isolation is shown [27]. Mitochondrial isolation can be performed in less than 30 min. Quality control parameters for mitochondrial isolation and transplantation have been established [28]

To meet the clinical needs of surgery, we have developed a rapid method for the isolation and purification of mitochondria that can be performed in less than 30 min (Fig. 3) [27]. The major benefits of this protocol are that it incorporates a standardized tissue dissociation process that allows for uniform and consistent homogenization of tissue that is not easily achieved with manual homogenization methods. In addition, we use differential filtration rather than centrifugation. The use of filtration eliminates time consuming and repetitive centrifugation steps allowing for more rapid isolation of highly purified, viable and respiration competent mitochondria.

In brief, two small pieces of tissue are collected from a non-ischemic source. For our experiments, we isolated mitochondria from the pectoralis major muscle. This mimics the surgical entry point via minimal thoracotomy where the pectoralis major muscle is close to the incision site and anatomically available. Alternative sources such as abdominal rectus muscle can be used if a sternotomy is performed. The muscle tissue is dissected free and two small pieces of tissue are obtained using a #6 biopsy punch. The tissue is homogenized in a volume of 5 mL of isolation buffer (300 mmol/L sucrose, 10 mmol/L HEPES–KOH, 1 mmol/L EGTA-KOH, pH 7.4) and then treated to 10 min of Subtilisin A enzymatic digestion on ice [27]. The digested tissue is then filtered through a series of filters (40, 40 and 10 uM), pre-wetted with isolation buffer, and the mitochondria are subsequently precipitated by centrifugation at 9×*g* for 10 min at 4 °C [27]. The exact isolation time is set by the 10 min enzymatic digestion and the final centrifugation. In our hands, this can be accomplished in less than 30 min. The mitochondria are counted by hemocytometer or by particle counter [27, 28]. The mitochondria are then suspended in freshly prepared Respiration Buffer [19, 20, 27].

In our myocardial protocols, the mitochondria are injected into the ischemic myocardium at 7–8 sites (0.1 mL per site) [19, 20]. We have observed that greater volumes 0.2 mL result in loss of mitochondria due to leakage at the site of injection. To inject the mitochondria we use a tuberculin syringe with a 28 G needle. No z-stich for wound closing is needed.

We also determine viability and purity in all mitochondrial preparations. Mitochondrial viability is determined by oxygen consumption rates using a Clark-type electrode and fluorescent probe analysis. Mitochondrial purity is determined firstly by light microscopy using fluorescent mitochondrial labels (MitoTracker CMXros) and then by transmission electron microscopy [19, 20, 27].

### The need for viable respiration competent mitochondria

We have investigated the efficacy of various sources of mitochondria. Our studies have demonstrated that there are differences in the absolute number of mitochondria that can be isolated from various tissue sources. Based on a constant starting weight the absolute number of mitochondria isolated in liver is greater than that obtained using skeletal muscle is greater than that obtained using atrial tissue [19]. Our studies demonstrate that there are no differences in the cardioprotective effects or cellular uptake of mitochondria isolated from various tissue sources [19, 20].

Mitochondrial sub-populations do not provide added cardioprotection. It has been demonstrated that in heart muscle there are differences in the oxygen consumption of the mitochondria dependent upon their location within the muscle tissue [29, 30]. To investigate the possible differential effects of the different sub-populations of mitochondria on cardioprotection, we have we have used total, interfibrillar and sub-sarcolemmal mitochondria isolated from the heart and mitochondria isolated from liver or skeletal muscle for mitochondrial transplantation. Our results demonstrate that mitochondrial transplantation provides similar cardioprotection regardless of their source [20].

Of prime importance in providing for efficacy of mitochondrial transplantation for cardioprotection is mitochondrial viability. The need for viable freshly isolated mitochondria is imperative. In our studies, we have shown that non-viable mitochondria, mitochondrial proteins, mitochondrial complexes, or mitochondrial RNA and DNA do not provide cardioprotection [20]. We also found that injection of exogenous ATP and ADP does not provide any protective effects on the heart, in agreement with studies from other investigators establishing that exogenous ATP supplementation and/or ATP synthesis promoters are ineffective in restoring high energy phosphate stores and provide no beneficial effects on post-ischemic functional recovery [31–33].

## Mitochondrial number

In studies performed to optimize the concentration of autologous mitochondria for cardioprotection, we have used 2 × 10^5^, 2 × 10^6^, 2 × 10^7^ and 2 × 10^8^ mitochondria per gram tissue (wet weight). The mitochondria were injected into the ischemic heart and infarct size was determined following 30 min temporary left anterior descending artery occlusion and 120 min reperfusion. The area at risk was 30 ± 4 %. Infarct size was determined to be 7.9 ± 2.9, 6.3 ± 3.1, 6.8 ± 2.6 and 6.0 ± 2.7 %, respectively for 2 × 10^5^, 2 × 10^6^, 2 × 10^7^ and 2 × 10^8^ mitochondria. No significant difference in infarct size was determined between groups. This was likely due to the small infarct size (7.9 ± 2.9 %) observed when using 2 × 10^5^ mitochondria per gram tissue wet weight. In all of our studies, we suspended the mitochondria in a final volume of 1 mL and inject at 8–10 sites within the area at risk. Mitochondrial concentrations >2 × 10^8^ were not entirely suspended in 1 mL buffer; so, these concentrations were not employed [personal communication McCully Lab].

Our studies suggest that the number of mitochondria needed for cardioprotection is not a function of the absolute number of mitochondria residing within the cell. In cardiomyocytes the mitochondria constitute 30 % of the total myocardial cell volume; however, only a small fraction of this number appears to be needed for cardioprotection following ischemia and reperfusion.

### Mitochondrial delivery

We have reported the delivery of mitochondria via direct injection. This route is simple and can be performed readily and efficiently using a tuberculin syringe with a standard 18 G needle [19, 20]. In cardiac procedures this approach allows for distribution without the need for additional suturing and injury to the myocardium. We have found that multiple 100 uL injection volumes remain within the tissue and no leakage is apparent [20].

In tissue cultures, we used co-incubation as a means for mitochondrial transfer. Cells are cultured in 24 well plates (5000/well) for 24 h in cell culture media. The media is then removed and freshly isolated mitochondria are resuspended in fresh cell culture media and then aliquots are layered onto the cells with the desired concentration of mitochondria (1 × 10^7^/well in 0.5 mL) [19, 34]. This method is effective and uptake is easily achieved.

Mitochondria can be pre-labelled prior to co-incubation. We have used MitoTracker Red CMX Ros (100 nmol/L), and other labels [19, 34]. In brief, mitochondria are labelled for 10 min at 4 °C and then the mitochondria are washed four times by centrifugation and resuspension in isolation buffer containing 300 mmol/L sucrose, 10 mmol/L HEPES–KOH, 1 mmol/L EGTA-KOH, pH 7.4 [19, 34]. In the last wash we keep the supernatant and use this in control samples to ensure that no free label remains and can be demonstrated in micrographs.

### Mitochondrial uptake

To determine the potential mechanisms by which mitochondria could be internalized by cardiomyocytes, studies were performed using specific blockers based on their wide use and established selectivity [34]. Our studies demonstrated that mitochondrial internalization by cardiomyocytes occurs through actin-dependent endocytosis [30]. Internalization of autologous mitochondria was not associated with tunneling nanotubes, caveola-dependent or clathrin-dependent endocytosis. The internalized mitochondria did not co-localize with lysosomal or autophagocytosis markers [34]. This is in agreement with the mechanism first proposed by Margulis [35], who hypothesized that the mitochondrion, existing as a prokaryotic cell was engulfed by a eukaryotic host cell and then was established within that cell. In vivo many methods of mitochondrial transfer between cells have been postulated; however, our observations show that externally presented mitochondria are readily internalized by a wide variety of host cells. This contrasts to intercellular mitochondrial transfer mechanisms where these organelles are transferred from one cell to another through cylindrical conduits (tunneling nanotubes) [36–39]. Although it is possible that intercellular mitochondrial transfer occurs after endosomal internalization, this particular mechanism does not seem to be involved with internalization of exogenous mitochondria.

### Mitochondrial distribution

In rabbit heart studies we have shown that injected mitochondria labeled MitoTracker Orange CMTMRos are viable and are visible at the site of the injection and are distributed >2–3 mm distant from the site of injection [19, 20].

The transplanted autologously-derived mitochondria are initially observed in the interstitial spaces surrounding cardiomyocytes, with extensive epicardial to sub-endocardial distribution. These transplanted mitochondria significantly increase tissue ATP content in the heart. At 1–2 h after injection, the transplanted mitochondria are detectable within cardiomyocytes and can be observed residing near the sarcolemma between Z-lines of the sarcomeres. Internalization into cardiomyocytes has been confirmed using mitochondria isolated from HeLa cells (human) in rabbit heart. The use of human mitochondria in the rabbit model allowed for the differentiation between native rabbit mitochondria and transplanted human mitochondria based on immune reactivity to a monoclonal anti-human mitochondria antibody [19, 20]. Transmission electron microscopy studies using immuno-gold staining verified that transplanted mitochondria are internalized into cardiomyocytes [19, 20].

In in vitro cardiomyocyte studies we have shown that the uptake of mitochondria is linear and time dependent. The transplanted mitochondria significantly increase (p < 0.05) cardiomyocyte ATP content as compared to control in both neonatal and adult cardiomyocytes [19, 20, 34].

### Immune and auto-immune reactions of mitochondrial transplantation

The transplantation of autogeneic mitochondria for the amelioration of myonecrosis offers a unique therapeutic potential. The isolation and preparation of autogeneic mitochondria from a patient’s own body would prevent inflammation and rejection. Our studies to date have demonstrated significantly decreased inflammatory markers (TNFα, IL-6, IL-10 MCP-1 and hsCRP) when compared to untreated regional ischemia [19]. Necropsy of hearts at 28 days after mitochondrial injection showed no inflammation or evidence of injury. At the same time, there was no increase in white blood cell counts in any animal receiving mitochondrial transplantation [19].

Multiplex analyses indicated that transplantation of autologous mitochondria significantly upregulated epidermal growth factor (EGF), growth-related oncogene (GRO), IL-6 and monocyte chemotactic protein-3 (MCP-3) expression. These cytokines have been shown to play key roles in angiogenesis, arteriogenesis, immunomodulation, progenitor cell migration, prevention of apoptosis and enhanced cell salvage and post-ischemic functional recovery (19). Previous studies have demonstrated that EGF plays an important role in ischemic injury protection in the heart through the stimulation of cell growth, proliferation, and migration [40–42]. GRO and Il-6 act together following infarction to improve cardiac function through the reconstitution of tissue mass. In this process IL-6 acts as a chemo-attractant which allows for enhanced vascularization, protection against cardiomyocyte apoptosis. These actions have been demonstrated to improve functional recovery in the heart [40, 42]. EGF, GRO and IL-6 act with MCP-3 to improve cardiac remodeling independent of cardiac myocyte regeneration [43].

On the other hand, no pro-inflammatory cytokines were upregulated by this treatment. There was no upregulation of cytokines associated with the immune response that is seen in patients with acute heart transplantation rejection (IL-1, IL-4, IL-6, IL-12, IL-18, IP-10, macrophage inflammatory protein (MIP-1α and MIP-1β) [19].

To test for autoimmunity we have used a specific assay to determine mitochondrial autoimmune response. The assay is used in the diagnosis of primary biliary cirrhosis and the complete assay is described [19]. In brief, serum is collected from test animals and is used for indirect immunofluorescent assay for the detection of antibodies directed against mitochondrial antigens (AMAs). Hep-2 cells are used as substrate. Human serum from a patient with primary biliary cirrhosis, containing a high titer of AMA, is used as a positive control. Diluted rabbit sera and human serum are incubated with the Hep-2 cells for 1 h at room temperature and the unbound antibodies are removed by three successive washes with PBS, Bound antibodies are detected with secondary antiserum to detect AMA [19]. Our results demonstrate that the injection of mitochondria, even at high concentrations 1 × 10^10^ does not induce any autoimmune response [19].

It is also of relevance that we did not find any signs of myocardial arrhythmia associated with mitochondrial transplantation. Our results, using serial long term ECG, QRS duration, and corrected QT interval, show no evidence of ventricular wall motion disturbances, left ventricle hypertrophy, valve dysfunction, fibrosis, or pericardial effusion at 4 weeks following transplantation of autogeneic mitochondria [19]. To ensure no arrhythmia was associated with mitochondrial transplantation we also performed optical mapping studies in the rat heart using 4.2 × 10^8^ mitochondria. The concentration of mitochondria was 8.4 × 10^7^/g tissue wet weight as compared to 2 × 10^5^ to 2 × 10^7^/g tissue wet weight, so that any acute arrhythmogenic responses would be observed. These studies failed to show any abnormal impulse propagation on the myocardial surface associated with mitochondrial transplantation.

### Mitochondrial alterations affecting cardioprotection

**Fig. 4:**
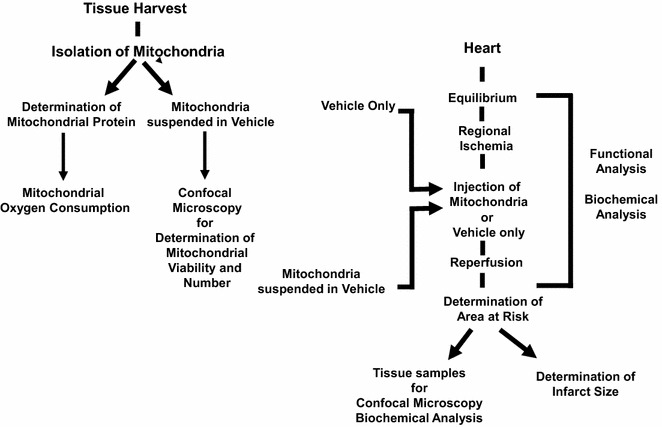
Experimental outline for mitochondrial transplantation studies [19, 20]

In our studies in the isolated perfused heart and the in situ blood perfused heart model we have followed a similar protocol (Fig. 4). In this protocol the heart is exposed using a mini-thoracotomy or a sternotomy. Muscle tissue from the pectoralis major tissue is harvested and used for mitochondrial isolation as described in Fig. 3. The heart is then made ischemic by temporarily snaring the left anterior descending artery to cease blood flow to the left ventricle. This is performed by passing a Prolene suture around the left anterior descending coronary artery with a cutting needle and passing both ends of the suture through a small vinyl tube to form a snare which was then pulled tight and fixed with a mosquito clamp [19, 20]. Following 29 min regional ischemia, hearts received eight, 0.1 mL injections delivered to the left ventricle of either vehicle alone or mitochondria suspended in vehicle. The mitochondria were injected into the epicardium using a tuberculin syringe with a 28 g needle. The snare was then released and coronary blood flow through the left anterior descending coronary artery was re-established. The hearts were allowed to recover and functional and biochemical data were collected. In the in situ blood perfused models we have used 4 weeks recovery [19]. In the isolated perfused heart 2 h reperfusion is used to allow for estimation of infarct size [20].

Once transplanted, mitochondria maintain viability and function, increasing ATP levels and ATP synthesis and, at least some of these remain in myocardial cells in situ for 28 days [19, 20, 34]. This is in contrast to present xeno- and allo-transplanted cells that provide limited improvement in heart function and are ultimately rejected leading to the loss of transplanted cells and recurrence of heart failure, despite the use of anti-rejection pharmaceuticals [44, 45].

The transplanted mitochondria also act to alter the myocardial proteome. Our studies in the in situ rabbit heart model show that there were distinct protein expression patterns between hearts receiving mitochondrial transplantation and control hearts receiving vehicle only. These changes occur rapidly after mitochondrial transplantation. At 10 min post injection we were able to detect changes in myocardial function by electrocardiogram and echo cardiographic analysis [19]. Myocardial tissue isolated from hearts treated with mitochondria transplantation showed significant alterations in the mitochondrial proteome at this time. Functional annotation clustering (*P* < 0.05, Enrichment Score > 2.0) indicated that protein pathways for the mitochondrion and the generation of precursor metabolites for energy and cellular respiration were significantly enriched in hearts receiving mitochondrial transplantation as compared to control hearts receiving vehicle only [19]. These changes in the proteome likely aided the long-term recovery of the myocardium through the up-regulation of mitochondrial associated pathways leading to enhanced high energy synthesis and mitochondrial repair.

In recent studies, we have shown that mitochondrial transplantation not only rescues cell function, increasing cellular ATP content and mitochondrial oxygen consumption, but also replaces damaged mitochondrial DNA [34]. These studies were performed in vitro using human HeLa cells deleted of mitochondrial DNA (*p*^0^ rho HeLa cells). HeLa *p*^0^ cells are depleted of mtDNA through serial passage with low concentrations of ethidium bromide [46]. HeLa *p*^0^ cells due to the deletion of mitochondrial DNA are incapable of synthesizing the mitochondrial encoded sub-protein constituents of the electron transport chain and as a result provide energy generation through fermentation but lack oxygen consumption capacity due to depletion of electron transport chain sub-proteins encoded by mtDNA. As a result of this inability to utilize the electron transport chain HeLa *p*^0^ cell mitochondria have no oxygen uptake and ATP synthesis is generated only by glycolysis. Our studies demonstrate that co-incubation of HeLa *p*^0^ cells with mitochondria isolated from HeLa cells containing intact mtDNA rescues HeLa *p*^0^ cell function by significantly increasing ATP content and oxygen consumption rates [34]. The enhanced intracellular ATP content was significantly increased in HeLa *p*^0^ cells following co-incubation with mitochondria at 24, 48, 72 h and 1 and 2 weeks [34]. Quantitative real-time RT-PCR analysis demonstrated that while there was no mitochondrial DNA in HeLa *p*^0^ cells, mitochondrial DNA was detected in HeLa *p*^0^ cells with internalized mitochondria from HeLa cells with intact DNA [34].These experiments demonstrated that mitochondrial transplantation can be used therapeutically to rescue cell function and replace damaged mitochondrial DNA.

### Mechanism of cardioprotection

The mechanisms through which the transplantation of autologous mitochondria provides for cardioprotection clearly involves both enhanced ATP production and alterations in the proteome, cytokine induction and replacement of damaged mtDNA. We speculate that these effects act to provide cardioprotection in two overlapping phases; (1): an immediate cardioprotective phase and (2): a late cardioprotective phase.

The immediate cardioprotective phase occurs during the first 10 min of reperfusion and is associated with transplanted mitochondria being closely associated with cardiac cells. This close association significantly enhances total tissue ATP and cardiac cell enrichment of differentially expressed proteins associated with mitochondrial pathways responsible for the generation of precursor metabolites associated with energy and cellular respiration. These modulations would allow for cardiac cells to respond to the ischemic insult during the immediate phase of reperfusion by providing needed ATP and needed up-regulation of mitochondrial pathways and as a consequence, would allow for cardiac cell rescue and enhanced post-ischemic functional recovery. These effects occurring during early reperfusion would then act in concert with late-cardioprotective effects to enhance long term viability and function.

The late-cardioprotective phase is evident prior to 1 h following mitochondrial transplantation. In this phase mitochondria are internalized into cardiac cells. ATP synthesis and oxygen consumption rate in cardiac cells is significantly enhanced. We hypothesize that it is during this late phase when the mitochondria are internalized by the cells that the mitochondria act to repair mitochondrial DNA damaged during ischemia. Support for this mechanism comes from our studies demonstrating that myocardial function is enhanced and myocardial infarct size is significantly decreased and that these alterations are evident and stable for at least 28 days post-injection, the duration of our current studies [19, 34].

### Mitochondrial transplantation in other organs

**Fig. 5:**
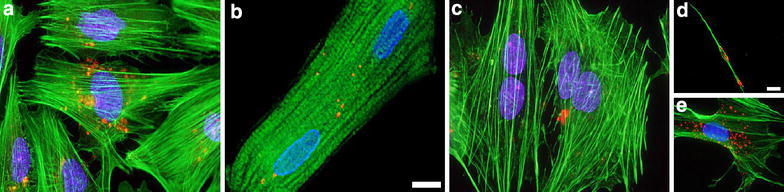
Transplanted mitochondria are internalized by a variety of cell types: **a** neonatal rat cardiomyocytes, **b** adult rat cardiomyocytes, **c** rat skeletal myoblasts, **d** embryonic rat neurons and **e** mixed embryonic rat neuronal cells. Internalized pHrodo Red-labeled rat liver mitochondria (*red*) following 4 h co-culture are shown in each cell type. In all images the *blue* stain is DAPI, alpha-actinin 2 is depicted in *green*. *Scale bars* are 10 um

Our studies have pioneered the efficacy of mitochondrial transplantation using direct tissue injection [19, 20]. While these studies have been focused mostly on the myocardium, we have also performed studies to show that mitochondrial transplantation can be performed in a variety of cell types using autogeneic, allogeneic and xenogeneic mitochondria (Fig. 5). Our studies demonstrate that mitochondrial transfer can be performed in neonatal and adult cardiomyocytes, skeletal muscle cells, human cells (HeLa, HeLa *p*^0^ vascular endothelial cells and mixed neuronal cell types [34]. The efficacy of uptake appears to be similar in all cell types we have examined with mitochondria being internalized in a time-dependent manner. Mitochondrial internalization in all cell types is evident following 1 h co-incubation and is significantly increased following 4 and 24 h co-incubation [19, 34]. The increase in mitochondrial internalization is correlated with increased cellular ATP content and cellular oxygen consumption [19, 34]. The uptake and action of mitochondria is similar to that obtained using microinjection. King and Attardi [47] have previously demonstrated that micro-injection of mitochondria into cell lines depleted of their own mitochondrial DNA allowed for the replacement of the resident mtDNA by exogenous mtDNA.

Mitochondrial transplantation is also efficacious in other disease models (Table 1). Elliott et al. [48] have recently used mitochondrial transplantation to enhance drug sensitivity in human breast cancer cells. The authors demonstrated that mitochondria isolated from human immortalized, untransformed mammary epithelia cell line, MCF-12A, and co-incubated with human breast cancer cell lines resulted in the uptake of untransformed cell line mitochondria inhibiting cancer cell proliferation and increasing the sensitivity of the cancer cells to anticancer agents.

**Table 1:** Mitochondrial transplantation

Tissue	Heart	Heart	Liver	Brain
Animal model	Rabbit	Rabbit	Rat	Rat
Experimental model	Langendorff perfused heart	In situ, blood perfused	In situ, blood perfused	In situ brain
Injury model	Ischemia/reperfusion	Ischemia/reperfusion	Ischemia/reperfusion	Parkinson’s disease
Mitochondria	Unmodified	Unmodified	Unmodified	Conjugated with PEP-1
Delivery	Direct injection	Direct injection	Direct injection	Direct injection
Outcome	Enhanced myocardial function following ischemia, enhanced cell viability	Enhanced myocardial function following ischemia, enhanced cell viability	Decreased liver tissue injury and apoptosis	Peptide-mediated mitochondrial transplantation lessened Parkinson’s disease movement disorder and attenuated the deterioration of dopaminergic neurons
Reference	McCully et al. [20]	Masuzawa et al. [19]	Lin et al. [51]	Chang et al. [50]

Chang et al. [49] has used peptide-mediated mitochondrial delivery to rescue cell function in cells harboring the mitochondrial DNA mutation MERRF A8344G. This methodology delivers isolated mitochondria to cells by first conjugating isolated mitochondria with Pep-1, a member of the cell-penetrating peptide family. Recently this same group has used this methodology to replace damaged mitochondria in a rat model of Parkinson’s disease [50]. The authors showed that Pep-1 conjugated mitochondrial transplantation into the midbrain of neurotoxin induced cells and Parkinson’s disease rat models significantly enhanced mitochondria function and partially ameliorated the degeneration of dopaminergic neurons.

Lin et al. [51] have shown that a splenic injection of isolated mitochondria significantly ameliorated liver ischemia/reperfusion injury in the rat, significantly decreasing serum enzyme markers of liver damage and liver cell apoptosis.

Kitani et al. [52] showed that isolated mitochondria are taken up and internalized by rat cardio-myoblasts and rescued mitochondrial respiratory function and improved the cellular viability.

The examples of the diseases and syndromes and/or clinical situations in which mitochondrial transplantation can be used are too many to discuss within this review. The effects of mitochondrial transplantation on mitochondrial disorders with associated heteroplasmy require more in depth analysis than has been currently provided. A key feature in this analysis is the source and use of the mitochondria. In cases such as ischemia/reperfusion injury or compartment injury to name just two, the use of autologous mitochondria would be effective only no alteration in the mitochondrial genome that could affect recovery was present. However, in the cases where mitochondrial DNA defects are present, the use of allo- and xeno-genetic mitochondria would be more appropriate. This would allow for the replacement of damaged mtDNA with intact functional exogenous mtDNA. At present there is not sufficient data for the recommendation of either allo- or xeno-genetic mitochondria as no immune or auto-immune data is available. The use of multiple treatments has not been reviewed nor has any toxicological analysis been performed as yet. Despite these obvious caveats, the therapeutic potential of mitochondrial transplantation is great and it is likely that this new treatment modality will significantly decrease morbidity and mortality associated with mitochondrial alteration or dysfunction.

## Conclusions

We have focused our research on myocardial ischemia and myocardial function in the adult heart; however, we have also shown that mitochondrial uptake occurs in neonatal cardiomyocytes, skeletal muscle, human (HeLa) cells and mixed neuronal cell cultures. The methodologies we present are uncomplicated and can be performed in any laboratory with limited investment. The major consideration for efficacy is that the mitochondria used in transplantation must be viable and intact to allow for uptake, internalization and efficacy.

The isolation and preparation of autogeneic mitochondria is rapid and purified mitochondrial are available within 30 min, a time-frame within the clinical time frame for use in surgery. The transplantation of mitochondria is not pro-arrhythmic and does not induce immune or auto-immune responses. Mitochondrial transplantation provides an efficacious cardioprotective protocol that can be used either as an exclusive intervention to ameliorate myonecrosis and enhance myocardial function or could be used as a primary intervention prior to subsequent auto-, allo- or xeno-geneic cellular regenerative interventions requiring extended times for cell isolation, purification and expansion.

The uptake of mitochondria appears to be universal. We foresee that mitochondrial transplantation will be a valued treatment in the armamentarium of all clinicians and surgeons for the treatment of varied ischemic disorders, mitochondrial diseases and related disorders.

All experiments described in this paper were approved by the Beth Israel Deaconess Medical Center Animal Care and Use Committee (032-2010, 065-2012), the Boston Children’s Hospital Animal Care and Use Committee (005-2013, 014-2013) and or, the Harvard Medical School Animal Care and Use Committee and conformed to the US National Institutes of Health (NIH) guidelines regulating the care and use of laboratory animals (NIH publication no. 5377-3, 1996). All research was performed in accordance to the American Physiological Society Guiding Principles in the Care and Use of Animals and the revised Animals (Scientific Procedures) Act 1986 in the UK and Directive 2010/63/EU in Europe.

## Abbreviations

ADP: adenosine di-phosphate
ATP: adenosine tri-phosphate
ANT: ATP/ADP translocator
DNA: deoxyribonucleic acid
EGF: epidermal growth factor
GRO: growth-related oncogene
IL-6: interleukin 6
IL-10: interleukin 10
MCP-3: monocyte chemotactic protein-3
Mi-CK: mitochondrial creatine kinase
mtDNA: mitochondrial DNA
ND 1-6: NADH dehydrogenase 1-6
TNFα: tumour necrosis factor alpha
VDAC: voltage dependent anion channel
